# Two-Step Resist Deposition of E-Beam Patterned Thick Py Nanostructures for X-ray Microscopy

**DOI:** 10.3390/mi13020204

**Published:** 2022-01-28

**Authors:** Javier Hermosa, Aurelio Hierro-Rodríguez, Carlos Quirós, María Vélez, Andrea Sorrentino, Lucía Aballe, Eva Pereiro, Salvador Ferrer, José I. Martín

**Affiliations:** 1 Departamento de Física, Universidad de Oviedo, 33007 Oviedo, Spain; uo232478@uniovi.es (J.H.); hierroaurelio@uniovi.es (A.H.-R.); quiroscarlos@uniovi.es (C.Q.); mvelez@uniovi.es (M.V.); 2 Centro de Investigación en Nanomateriales y Nanotecnología (CINN), CSIC-Universidad de Oviedo, 33940 El Entrego, Principado de Asturias, Spain; 3 ALBA Synchrotron, 08290 Cerdanyola del Vallès, Spain; asorrentino@cells.es (A.S.); laballe@cells.es (L.A.); epereiro@cells.es (E.P.); ferrer@cells.es (S.F.)

**Keywords:** e-beam lithography, magnetic nanostructures, transmission X-ray microscopy

## Abstract

Patterned elements of permalloy (Py) with a thickness as large as 300 nm have been defined by electron beam lithography on X-ray-transparent 50 nm thick membranes in order to characterize their magnetic structure via Magnetic Transmission X-ray Microscopy (MTXM). To avoid the situation where the fragility of the membranes causes them to break during the lithography process, it has been found that the spin coating of the resist must be applied in two steps. The MTXM results show that our samples have a central domain wall, as well as other types of domain walls, if the nanostructures are wide enough.

## 1. Introduction

Nowadays, there is wide interest in the exploration of the 3D magnetization configuration of patterned magnetic structures [1,2], as some of these structures might be topologically protected, which is important for subsequent applications. The interest in the study of microscopic magnetic structures lies in the possibility of the development and fabrication of sensors for numerous applications, including optics, memory devices, and biology [3,4,5,6,7]. On the other hand, it is important to note that the three-dimensional aspect ratio of the patterned structures usually implies a large thickness of the material.

Additionally, once the patterned elements with 3D magnetic configuration have been obtained, it is necessary to perform their appropriate characterization. In this sense, among other methods, Magnetic Transmission X-ray Microscopy (MTXM) has been shown to be a suitable technique to analyse the magnetic structure, as it provides a high spatial resolution of the sample, as well as element-specific magnetic information [8]. In addition, the data acquired with MTXM can be later processed by tomographic methods in order to obtain a magnetic tomographic reconstruction, which can allow us to resolve the three-dimensional magnetization within the sample volume [9]. In fact, transmission methods are excellent for the visualization of magnetic states in a sample, while they require probes with high enough penetration depths [10]. Thus, in order to take advantage of the MTXM technique, it is mandatory to use membrane substrates with low X-ray absorption. In particular, the membranes must be thin enough to let through a considerable amount of light, since a large signal-to-noise ratio is crucial for these transmission measurements [11]. Then, completing the patterning process on top without damaging them becomes a challenge. While the magnetic properties of Py films can vary depending on the substrates [12], we have seen that our nanostructures share the same behaviour when they are patterned on the membranes or on the Si substrates.

In this work, the electron beam lithography (EBL) process for MTXM membranes is described. In particular, the article is focused on how the fabrication of large-thickness elements (permalloy microstructures) drives the division of the spin coating of the resist in two steps, which helps to avoid the membrane breaking during the patterning process. In this way, several series of lithographed structures have been patterned on the membranes, and the MTXM results of some of them that we already measured at the MISTRAL beamline of the ALBA synchrotron will be shown.

## 2. Materials and Methods

**Figure 1:**
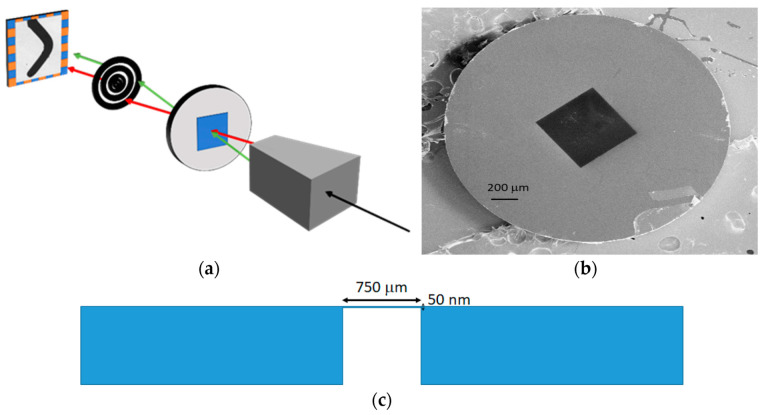
(**a**) Sketch of the MTXM system. From right to left: X-rays condenser, sample, Fresnel zone plate, and detector. (**b**) SEM image of a Si_3_N_4_ membrane. The black square is the 50 nm thick Si_3_N_4_ membrane. (**c**) Schematic cross-section view of a membrane.

Several nanostructures have been made using electron beam lithography (EBL) on membrane substrates. EBL is a method that uses a tightly focused beam of electrons scanned over the surface of the substrate. The samples include Si_3_N_4_ transparent membranes for MTXM measurements, with a window area of 750 μm × 750 μm and a very small thickness of 50 nm, where the nanostructures must be patterned (Figure 1). In the measurements, the focused X-rays—which have a fixed energy—impact on the sample, before passing through the membrane and creating the image in the detector after being diffracted by a Fresnel zone plate.

In order to perform the EBL process, it is necessary beforehand to extend a resist over the sample (250 ± 20 nm). In our case, a PMMA 950K A4 resist from Microchem is used, which is spin coated at 4800 rpm for one minute, and then it is baked at 150 °C in an oven for more than 2 h. After the exposure of the pattern in a scanning electron microscope, the development of the resist (≈45 s) takes place. Due to the thinness of the membrane, the whole process must be performed very carefully in order to avoid any possible break. In our case, each pattern has several elements. In particular, the nanostructures are designed to have a curved (hyperbolic) shape to easily set a magnetic domain wall in the bent region after the application of an external magnetic field.

After the development, a permalloy (80% Ni and 20% Fe alloy) is deposited by sputtering at an Ar working pressure of 3 × 10^−3^ mbar, as reported before [10], to obtain smooth surfaces. One remarkable characteristic of Py is that it is a soft magnetic material, and, therefore, its magnetic properties can be controlled by structural engineering on the nanoscale that results in a variety of behaviours [13,14,15]. The key to the process is the Py thickness. To contain 3D magnetic configurations, it must be wide and thick enough to leave some room for the formation of complex domain walls; otherwise, only ordinary walls (transverse/vortex walls, typically appearing in thin films) would be present, as there would not be enough space in the nanostructures to allow the creation of more complex walls. In fact, according to the characterization that we have carried out via Magnetic Force Microscopy (MFM) in structures patterned on Si substrates, we have determined that a width of at least 1 μm is needed. Finally, lift-off with acetone is performed to remove the remaining resist, leaving Py only in the already-exposed zone.

Gold particles with a diameter of 100 nm were sprinkled onto the sample to favour the alignment of the images acquired with MTXM. Element-specific magnetic Transmission soft X-ray Microscopy (TXM) imaging of the Fe absorption energy (706.8 eV) has been performed with the microscope installed at the Mistral beamline of the ALBA synchrotron [16]. There, X-ray Magnetic Circular Dichroism (XMCD), which consists of a magnetic material absorbing X-rays differently according to the helicity of the circularly polarized X-rays, is used [17].

## 3. Results and Discussion

**Figure 2:**
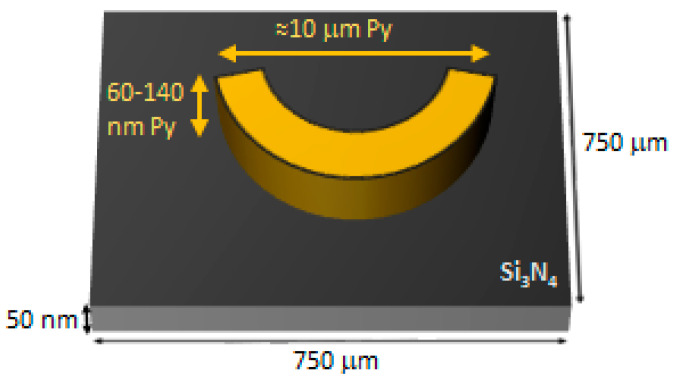
Sketch of the membrane with a Py strip (“boomerang”).

**Figure 3:**
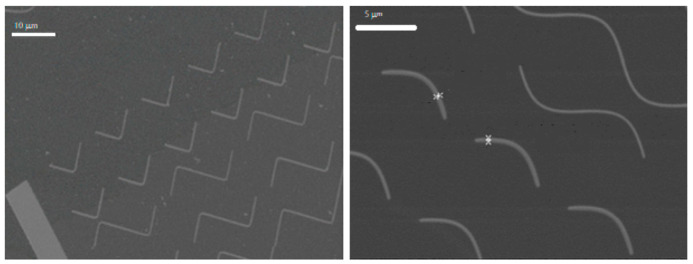
SEM images of some 100 nm thick Py nanostructures patterned on MTXM membranes. In the right picture, the width of the two highlighted “boomerangs” is about 425 nm.

The purpose of this research is to look for the appropriate curvature and the suitable electronic dose to obtain the desired magnetic walls. To perform this efficiently, several arrays of nanostructures are disposed in every membrane, so we have in each sample different curvatures and different electronic doses, the highest dose being around one hundred times higher than the lowest one. This layout gives a great variety of structures. The nanostructures have a curved shape similar to a boomerang, as sketched in Figure 2. The “boomerang” widths range from 0.1 μm to 2.5 μm in the lithographed samples, whereas they have an approximate length of 8–12 μm, depending on the curvature and on the Py thickness. An example is shown in Figure 3, where SEM images of some 100 nm thick Py nanostructures can be seen.

**Figure 4:**
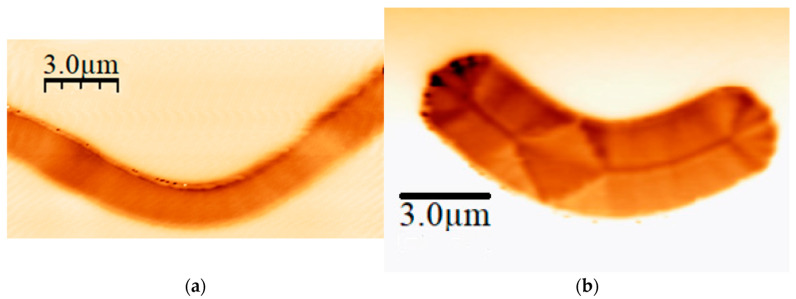
MFM images of: (**a**) 80 nm Py “boomerang”; (**b**) 140 nm Py “boomerang”.

As was indicated in Section 2, we have performed Magnetic Force Microscopy measurements in samples grown on Si substrates, which have different Py thicknesses. In view of the MFM images, a wide “boomerang” is needed in order to see special features in the magnetization. As it can be seen in Figure 4, in an 80 nm Py “boomerang”, the magnetization just “goes” from one end of the strip to the other, while different types of domain walls can be seen in a 140 nm Py “boomerang”. That is the reason to increase the Py thickness. Unfortunately, due to the characteristic shape of the membranes and their inherent fragility, breaks are not unusual. In fact, Py layers of up to 100–120 nm were made with relative ease, but the common method seemed not to be the proper way to deal with samples with thicker Py layers, since all of them broke. In fact, the aim is to have a good aspect ratio in the thickness axis and, therefore, a larger resist thickness in the lithography process is needed.

**Figure 5:**
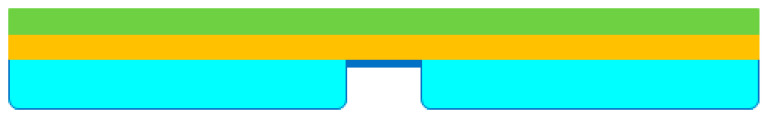
Schematic view of the two layers of PMMA resist on the MTXM membranes.

**Figure 6:**
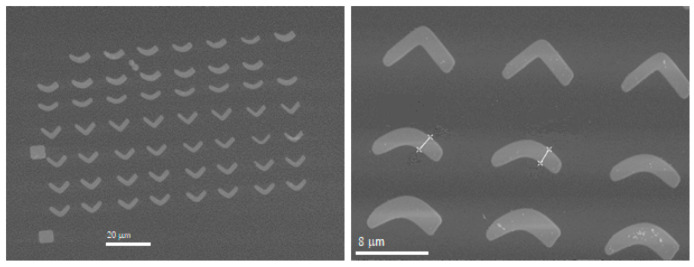
SEM images of some 140 nm thick Py nanostructures. In the right picture, the width of the two highlighted “boomerangs” is about 2100 nm.

In light of this problem, a different approach was used. Instead of depositing a thick layer of resist with the total thickness needed for the lithography process (which drives the breaking of the membranes), two thinner PMMA 950K A4 resist layers (each one with 250 nm in thickness) were consecutively extended and baked as sketched in Figure 5. By doing so, it is possible to make thicker samples. This result indicates that the deposition and baking of the first PMMA layer improves the mechanical response of the membrane before the deposition of the second layer and, therefore, it does not break in the second step. The width rises from about 400 nm to more than 2 μm, as can be seen in Figure 6, hence giving more space for domain walls to form.

**Figure 7:**
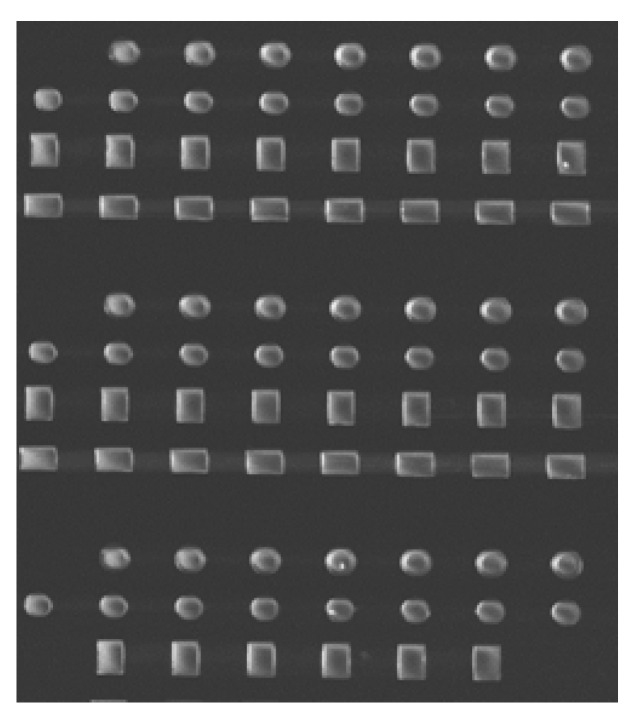
SEM image of 300 nm thick Py nanostructures. The size deviation is lower than 5%.

In fact, with this new method to prepare the PMMA resist on the 50 nm thick MTXM membranes, it is even possible to pattern Py elements with a thickness up to 300 nm, as shown in Figure 7.

**Figure 8:**
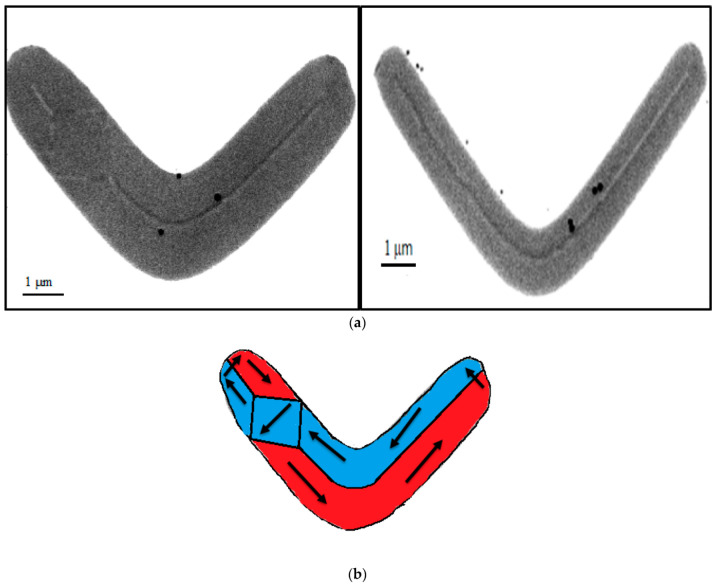
(**a**) MTXM image of two Py nanostructures. A central domain wall can be seen in both of them, as well as a diamond state in the left one. Black dots are gold nanoparticles for microscopy alignment (100 nm of diameter). (**b**) Sketch of the in-plane magnetization in a Py nanostructure with a central wall and the diamond state.

Once the patterned samples have been obtained, a magnetic field has been applied to them to favour the appearance of domain walls, and then MTXM measurements at the MISTRAL beamline in the ALBA synchrotron have actually been performed with several Py nanostructures. The measurements, as depicted in Figure 8, show the appearance of several domain walls. In fact, a central domain wall extends all over each sample, showing black-and-white contrast colours. That means different out-of-plane magnetization orientations, because these images were collected at a normal incidence. Besides the central wall, a rhomboid structure appears in wide structures, where the wall bifurcates. Further analysis, out of the scope of this work, will be needed to see what types of domain walls are present in these samples and what their microscopic configuration is.

## 4. Conclusions

The results of this work show how the spin coating resist thickness is a key factor in the fabrication of micro- and nanostructures on fragile MTXM Si_3_N_4_ membranes (50 nm thick) when analysing their microscopic 3D magnetic configuration. In fact, the new method applied to these nanostructures indicates that the spin coating in two steps allows a successful lift-off process when the membranes are used in the experiment, and it is even possible to obtain 300 nm thick patterned elements. In fact, it has been already possible to characterize, at normal incidence, some of the patterned elements via MTXM measurements, which shows that the formation of domains and domain walls is actually favoured by the width and thickness of the structure.
